# Transcriptome analysis reveals molecularly distinct subtypes in retinoblastoma

**DOI:** 10.1038/s41598-023-42253-4

**Published:** 2023-09-30

**Authors:** Qi Zeng, Sha Wang, Lu Chen, Jinwei Wang

**Affiliations:** 1 Hunan Provincial People’s Hospital (The First-Affiliated Hospital of Hunan Normal University), Changsha, 410005 China; 2 Eye Center of Xiangya Hospital, Central South University, 87 Xiangya Road, Changsha, 410008 China; 3 Hunan Key Laboratory of Ophthalmology, 87 Xiangya Road, Changsha, China; 4 National Clinical Research Center for Geriatric Disorders, Xiangya Hospital, Central South University, Changsha, China

**Keywords:** Cancer genetics, Eye cancer, Oncology

## Abstract

Retinoblastoma is the most frequent intraocular malignancy in children. Little is known on the molecular basis underlying the biological and clinical behavior of this cancer. Here, using gene expression profiles, we demonstrate the existence of two major retinoblastoma subtypes that can be divided into six subgroups. Subtype 1 has higher expression of cone related genes and higher percentage of RB1 germline mutation. By contrast, subtype 2 tumors harbor more genes with ganglion/neuronal features. The dedifferentiation in subtype 2 is associated with stemness features including low immune infiltration. Gene Otology analysis demonstrates that immune response regulations and visual related pathways are the key molecular difference between subtypes. Subtype 1b has the highest risk of invasiveness across all subtypes. The recognition of these molecular subtypes shed a light on the important biological and clinical perspectives for retinoblastomas.

## Introduction

Retinoblastoma, the most common pediatric intraocular cancer, is a rare childhood cancer of the developing retina, occurring in approximately 1 in 17,000 live births^1–3^. The primary objective of retinoblastoma treatment is to save the child's life by detecting the tumor early, treating it, and preventing metastasis^2–4^. Preserving the eye and maximizing visual potential is a secondary treatment goal^5^. Retinoblastoma is linked to low patient survival in low-income countries due to delayed diagnosis, limited access to specialized retinoblastoma care, and socioeconomic factors^6^. Tumor remission is achieved in over 95% of cases in high-income countries, but some patients still develop metastases, which can spread through the optic nerve into the central nervous system or through the sclera to the orbit^7,8^. Therefore, it is crucial to identify progressive retinoblastoma early to begin treatment. A few invasive gene signatures may serve as predictive biomarkers for retinoblastoma, demonstrating the molecular heterogeneity within the disease^9^.

Retinoblastoma is typically caused by the RB1 tumor suppressor gene becoming inactive on both alleles^8,10^. A small percentage of retinoblastomas that are not inherited (less than 2%) are initiated by MYCN-amplification without RB1 inactivation^11^. While hereditary retinoblastomas tend to be bilateral, non-hereditary cases are usually unilateral. The human retina consists of six types of neurons (rod and cone photoreceptors, bipolar, amacrine, horizontal, and ganglion cells) as well as Müller glia, all of which originate from retinal progenitor cells that can produce different cell types^12^. Some studies have suggested that the cell-of-origin for retinoblastoma is a cone precursor, while more recent research has suggested that certain retinoblastoma cells may have originated from ganglion cells^2,13^. However, it is unclear whether the cell-of-origin of retinoblastoma is linked to the progression and spread of the cancer.

A few attempts have been made to unveil the heterogeneity of retinoblastoma regarding the genetics and molecular expression other than RB1^10,14,15^. Copy number alteration (gains of 1q, 2p, 6p and losses of 13q and 16q) is regarded with the retinoblastoma recurrence^16,17^. A retinoblastoma invasion gene signature demonstrated its predictive capacity for retinoblastoma progression. Several studies have reported a strongly positive correlation between high copy-number alterations, age at diagnosis, and other clinical status with low differentiation of retinoblastoma, highlighting the subtypes exist in the retinoblastoma^18–20^. Despite these findings, there still is no consensus on the association of molecular features with the clinical phenotypes for retinoblastoma.

In this work, we identify two major subtypes of retinoblastoma associated with different by investigating 114 retinoblastoma gene expression. Within each subtype, we also identify several subgroups (four subgroups in subtype 1 and two subgroups in subtype 2). Consistent with previous studies, one subtype (subtype 1) has a higher score of cone marker while another subtype (subtype 2) has a relatively higher score of ganglion marker. Each subgroup has distinct profiles of retinal markers, stemness expression, highlighting the different cell-of-origin in retinoblastoma. Besides retinal markers and Photoreceptor gene expression, we also find the immune response between subtypes of retinoblastoma is distinct. Of them, subtype 1b has higher proportion of immune cell infiltration (including neutrophils, cytotoxic lymphocytes, monocytic lineage and myeloid dendritic cells) and a higher score of invasive signature.

## Methods

### Patient cohort

This cohort comprised 114 retinoblastoma cases at diagnosis who presented to Eye Center of Xiangya Hospital between 2018 and 2021. This study is conducted in accordance with Declaration of Helsinki. All subjects gave All subjects gave written informed consent in accordance with the Declaration of Helsinki. The samples were used under a protocol approved by the Institutional Review Board of Human Subjects Research Ethics Committee of the Eye Center of Xiangya Hospital, China.

### Gene expression profiles and subtyping

Gene expression profiling was performed in this cohort of 114 retinoblastoma using Affymetrix Human Genome U133 Plus 2.0 Array. Briefly, the gene-wide expression was acquired by the GeneChip Scanner 3000 and the expression matrix by probe was annotated by the package “hgu133plus2.db”. The expression matrix per gene was corrected by “removeBatchEffect” to remove batch effect. Principal component analysis was employed for dimension reduction exploration. The distances of samples were determined by the root-mean-square deviation of the top 2000 genes. For unsupervised clustering, we performed hierarchical clustering with agglomerative average linkage in this study to detect the robust clusters. Distance metric 1 − (Pearson’s correlation coefficient) was utilized for variances detection between samples. All subtype investigation was conducted by the package “ConsensusClusterPlus”.

### Differentially expressed gene and pathway analysis

Differentially expressed gene (DEG) analysis was conducted for identification of featured genes on the major subtype level. DEG was performed on linear modelling of indicated (co-) variates on expression values by limma^21^. p-values generated from limma modelling were corrected for multiple hypothesis testing by Benjamini and Hochberg false discovery rate (FDR) adjustments. Each subtype was tested against another subtype to generate this subtype featured genes. The FDR-adjusted p-values < 0.01 and |log Fold Change (FC)| > 1.5 were considered statistically significant. The DEGs between subtypes were visualized by heatmaps.

The pathway enrichment analysis was performed by “clusterProfiler”. Briefly, DEGs for subtype from previous analysis were subjected to the gene ontology analysis. Adjusted p value was calculated by Bonferroni-Holm methods and adjusted p value less than 0.01 was regarded as the significantly enriched pathway. Gene sets tested (HALLMARK) were from the Molecular Signatures Database (MSigDB).

### Retinal markers and stemness

We examined the expression level of retinal markers (including cone markers and ganglion markers, as described in a previous study) in each subtype. Stemness feature was assessed by the stemness index^22^. Kruskal–Wallis and Wilconxon test was performed to examine the statistical significance between two groups and multiple groups, respectively. A p-value less than 0.05 was considered as the statistical significance.

### Immune infiltration patterns

We employed the Microenvironment Cell Populations-counter (MCPcounter) to investigate the relative abundances of the immune cell infiltrating retinoblastoma^23^. Immune cell proportions were compared in each subgroup of retinoblastoma.

### Progressive retinoblastoma gene signature

We examined the expression of progressive retinoblastoma gene signature in each subgroup. As described before, the progressive retinoblastoma gene signature contained CLUL1, CNGB1, ROM1 and RDH12^9^. We compared these gene signature in each subgroup individually and the meta score of these gene signatures. Meta-scores were calculated as the average expression of the genes involved and then centered and scaled.

## Results

### Identification of molecular subtypes in retinoblastoma

**Figure 1:**
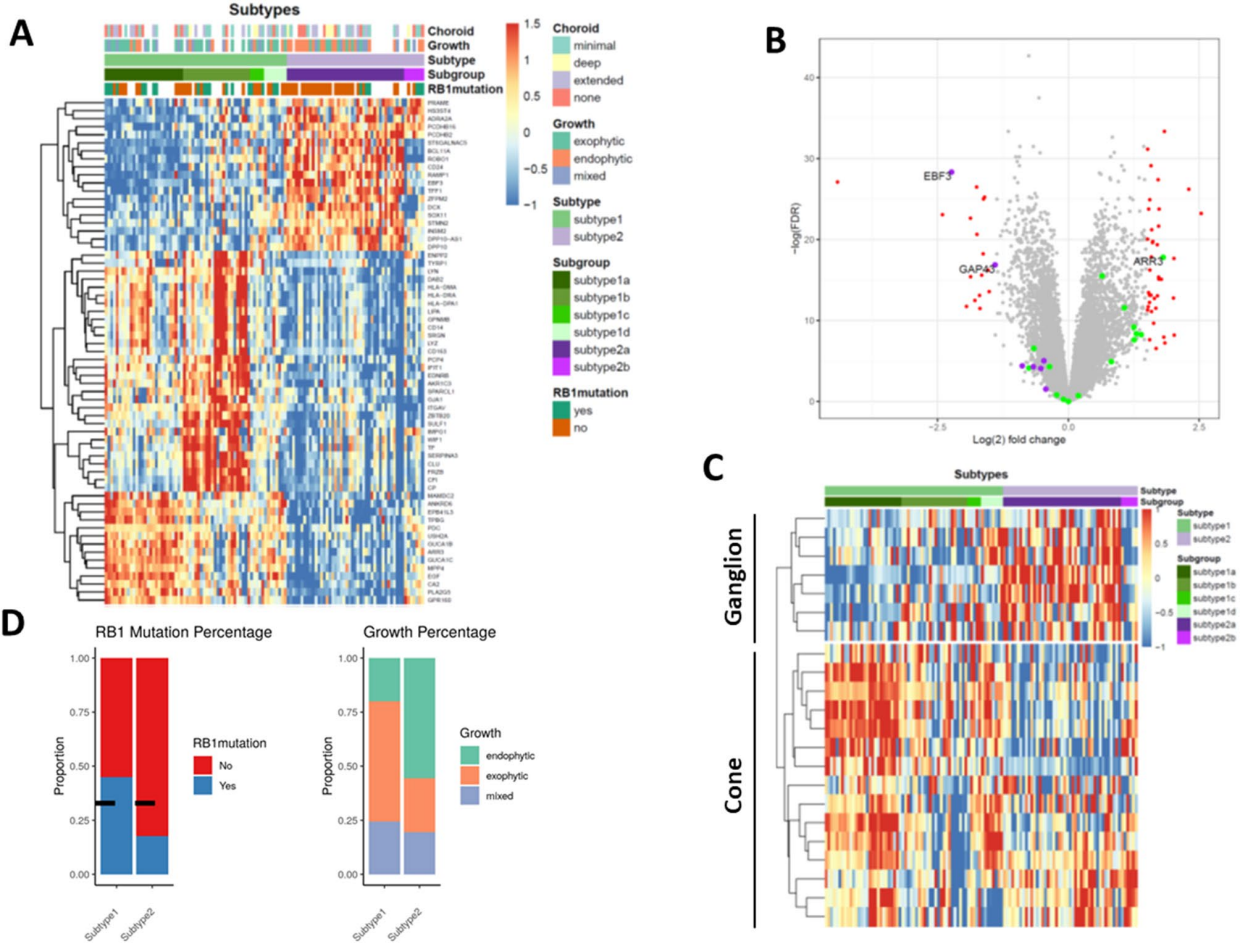
Consensus clustering showing two major subtypes and six subgroups in retinoblastoma. (**A**) Heatmap showing the different expressed genes between subtypes. (**B**) Volcano plot showing the different expressed genes between two subtypes. Red dots, different expressed genes; Purple dots, ganglion related genes; Green dots, cone related genes. (**C**) Heatmap showing the cone and ganglion genes between subtypes. (**D**) Barplot showing the percentage of RB1 germline mutation and Growth type between two major subtypes.

To investigate the different retinoblastoma molecular subtypes, we analysed the transcriptomes of 114 retinoblastoma. Consensus clustering of all samples were used for the subtyping of retinoblastoma. Consensus average linkage hierarchical clustering of 114 samples identified two clearly robust subtypes with clustering stability increasing. We also noticed there was a heterogeneity within the two subtypes and consensus matrix showed a clear separation between subgroups and there was a minimal overlap across subgroups when k = 6 (Supplementary Fig. 1). Notably, subtype 1 can be categorized into four subgroups (subtype 1a, subtype 1b, subtype 1c and subtype 1d) while subtype 2 was divided into two subgroups (subtype 2a and subtype 2b) (Supplementary Table 1). We then queried the differentially expressed genes (DEGs) between subtypes to uncover the biological differences. A total of 56 genes were identified as DEGs between subtype 1 and subtype 2 (Supplementary Table 2). Of them, 39 genes were up-regulated in the subtype 1. Of these 39 subtype 1 genes, we can still find the differences between subgroups. Photoreceptor genes (ARR3, GUCA1B) were relatively enriched in subtype 1a and subtype 1d, while WNT pathway related gene (WIF1) were relatively high in subtype 1b (Fig. 1). Subtype 1c had the moderate expression of all subtype 1 genes. We also noticed a high level of immune response genes (CD14, CD163, HLA-DMA, HLA-DRA and HLA-DPA1) were presented in the subtype 1b, suggesting this subgroup could have a higher immune response and immune cell infiltration. Within subtype 2, subtype 2a and 2b had a higher expression of ganglion genes (EBF3 and GAP43) but subtype 2b has a relative low expression of neurodevelopment genes (DCX, ROBO1, ST6GALNAC5, TFF1). Our subtyping was also consistent with the previous study, where one subtype 1 and 2 had a higher expression of cone and ganglion markers, respectively (Fig. 1).

We also noticed that higher percentage of RB1 germline mutation was observed in the Subtype1, especially in Subtype 1b (Fig. 1A,D), as compared with Subtype2. Exophytic growth was predominately found in Subtype1 while endophytic growth was the major growth type in subtype2 (Fig. 1D). We also noticed that young patients (< 24 months) were predominately in Subtype1, especially in Subtype1a and 1b. More female patients were classified into subtype 2a (Supplementary Table 3).

**Figure 2:**
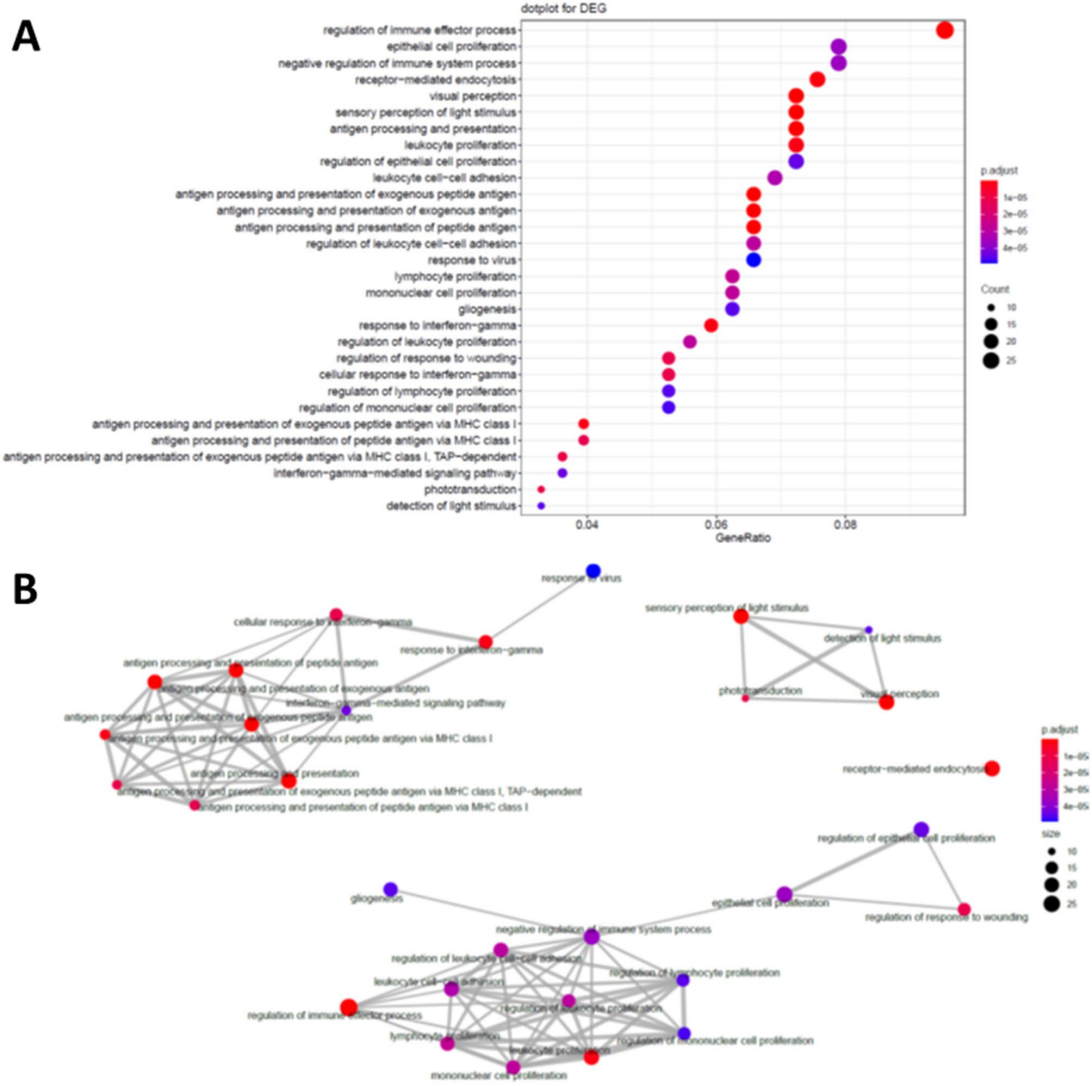
Gene Ontology analysis of the different expressed genes between subtypes. Dotplot (**A**) and Emap plot (**B**) showing the enriched pathways of the different expressed genes.

To further explore the difference of biological function between subtypes, we then investigated the biological pathways by DEGs. Dotplot for DEGs shows immune response, epithelial cell proliferation and visual perception were the most significantly different between subtype 1 and 2 (Fig. 2). This result was confirmed by the emap plot, where the significant pathways were categorized into three main clusters. These results suggested that the biological difference between subtypes of retinoblastoma were mainly in the immune response, visual perception and epithelial cell proliferation.

### Subgroups of retinoblastoma shows distinct profiles regarding retinal markers and stemness

**Figure 3:**
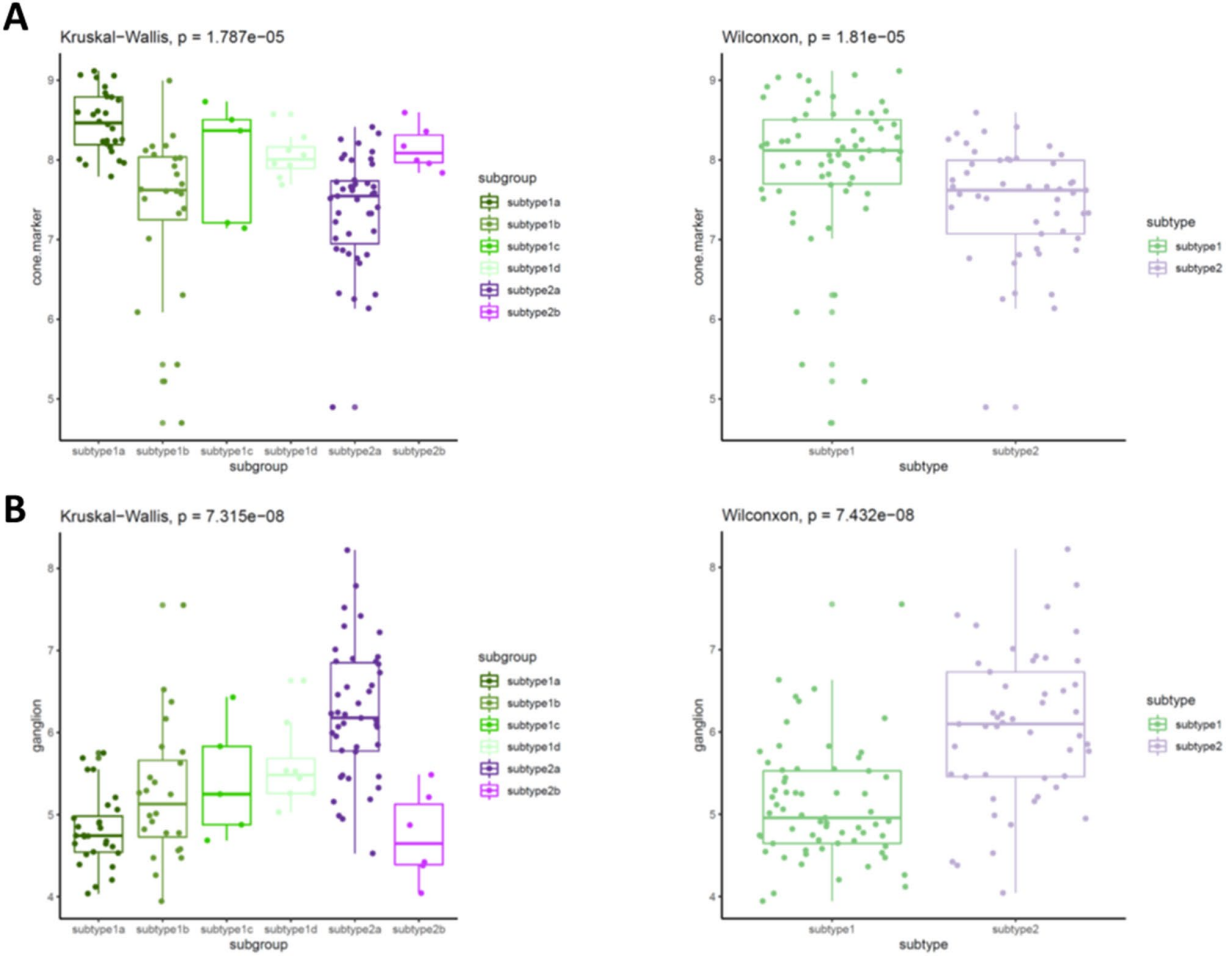
The cone and ganglion expression between subtypes. (**A**) Boxplot showing the expression of cone related genes in subtypes. (**B**) Box plot showing the expression of ganglion related genes in subtypes.

Since the different subtypes of retinoblastoma had distinct activation of biological pathways, we then examined whether these retinal markers maintained the same levels across subgroups. We investigated that the meta score for cone markers and ganglion markers in subtype 1 and 2, respectively. As expected, subtype 1 had a higher meta score of cone markers (Wilconxon test, p = 1.81 × 10^−5^) while subtype 2 had a higher level of ganglion meta score (Wilconcon test, p = 7.432 × 10^−8^) (Fig. 3). Interestingly, these cone and ganglion markers did not remain the same levels within the same subtypes. Subtype 1ahad the highest meta score for cone markers and subtype 1b showed the lowest meta score in all four subtype 1 subclasses. Subtype 2ahad the lowest cone meta score but subtype 2b had the similar score to subtype 1. For ganglion score, subtype 1d had the highest of all four subtype 1 subclasses and subtype 2ahad a significant higher meta score across all subgroups. Similarly, subtype 2b had the lower meta score of ganglion markers as compared with subtype 2abut the comparable meta score as compared with the subgroups in subtype 1, suggesting subtype 2b might originated from cone rather than ganglion. These results highlighted the heterogeneity of subtypes in retinoblastoma in terms of tissue of origin and classifying the subtypes of retinoblastoma by the tissue of origin need to be justified.

**Figure 4:**
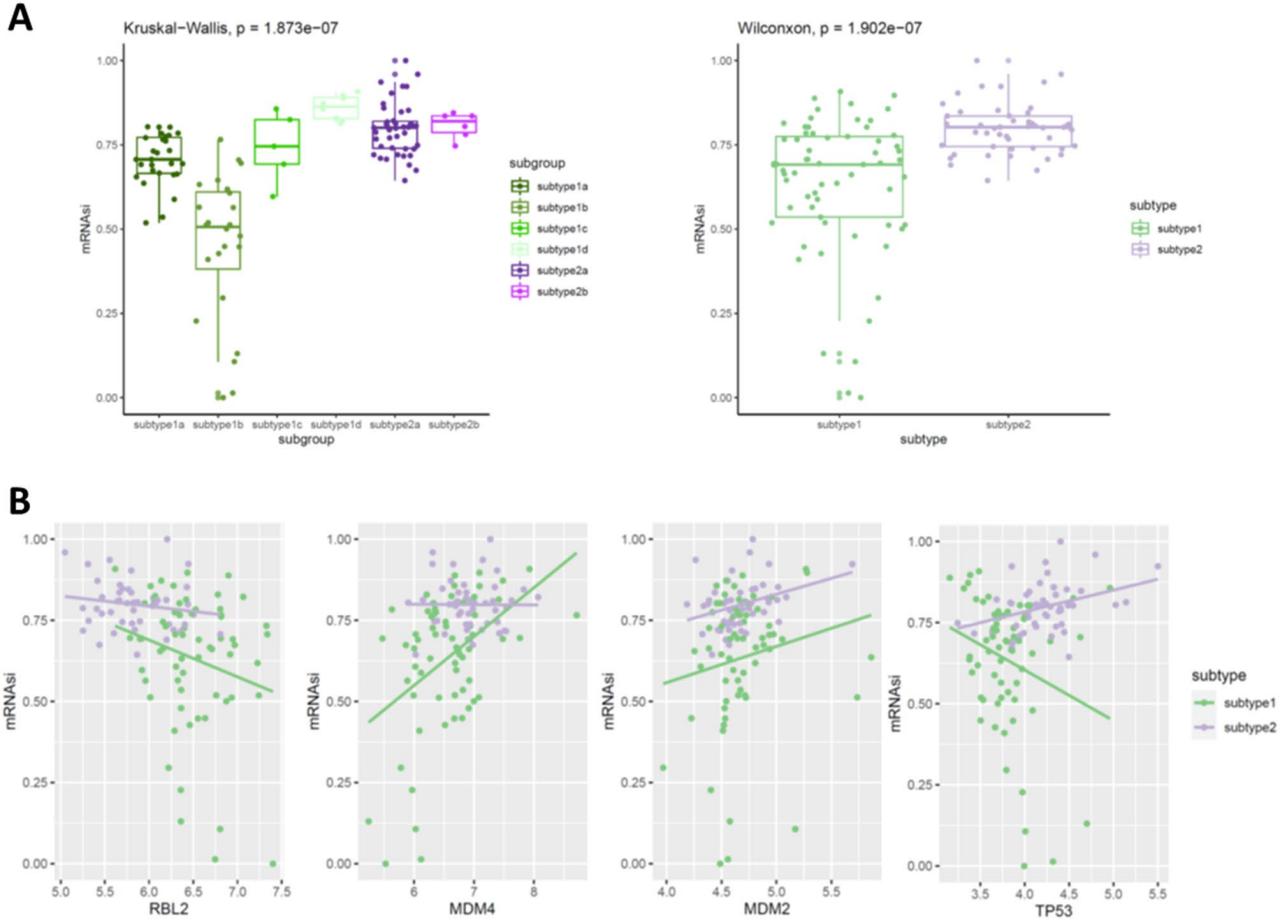
Stemness index between subtypes. (**A**) Boxplots showing the stemness index between subtypes. (**B**) Dot plots showing the correlation of stemness index with RBL2 and TP53 related genes between subtypes.

We then tested whether the stemness was different in subtypes of retinoblastoma. Subtype 2 had a higher stemness index than subtype 1, suggesting subtype 2 likely had more undifferentiated cells. In subtype 1, samples with low stemness index were enriched in the subtype 1b, suggesting subtype 1b likely contains more differentiated cells (Fig. 4). Then, we queried the RB and TP53 related genes expression to test the correlation of them with stemness feature across subtypes because a decrease of RBL2 and TP53 was observed in neuronal/ganglion subtype of retinoblastoma. Interestingly, we found RBL2 and TP53 relevant genes (MDM4, MDM2 and TP53) were slightly higher in subtype 2 retinoblastoma than these in subtype 1. In subtype 1, stemness index was negatively correlated with RBL2 and TP53 but showed a strong correlation of MDM4. In subtype 2, there was no strong correlation of these genes with stemness index (Fig. 4).

### A large difference of immune infiltration patterns between subtypes of retinoblastomas

**Figure 5:**
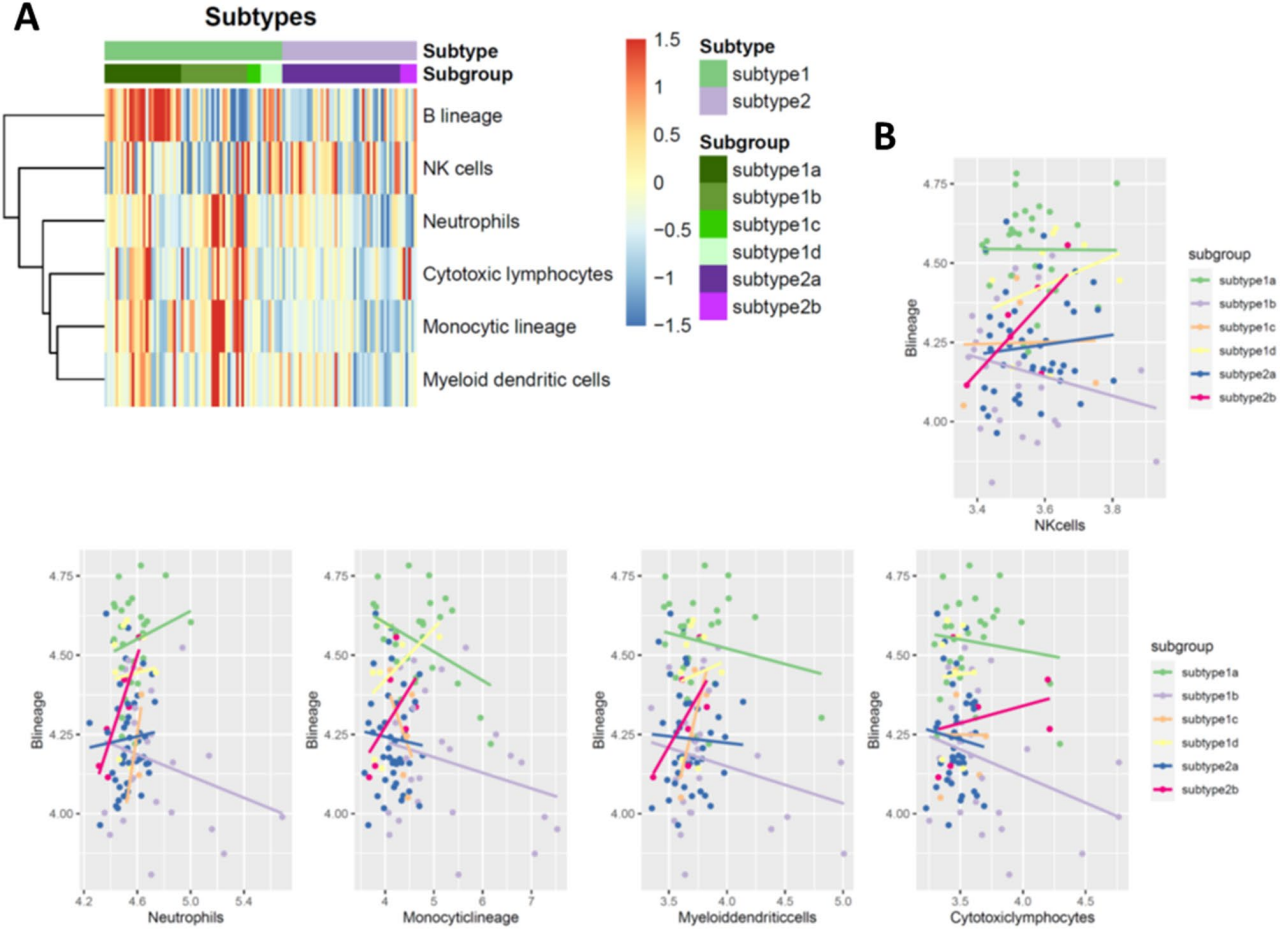
Immune cell infiltration between subtypes in retinoblastoma. (**A**) Heatmap showing the immune cell infiltration between subtypes. (**B**) Dot plots showing the correlation of B lineage proportion with other immune cells across subtypes.

Since the pathway analysis of retinoblastoma subtypes indicate the immune response was the one of the key features, we next investigated the immune cell infiltration in each subtype of retinoblastoma. Through MCPcount algorithms, the percentages of different immune cell lineages across subtypes of retinoblastoma were predicted. Notably, a higher B cell lineage was only observed in subtype 1a and high proportion of neutrophils, cytotoxic lymphocytes, monocytic lineage and myeloid dendritic cells were presented in subtype 1b (Fig. 5). In subtype 2, the proportion of all immune cells lineages were relatively low as compared with subtype 1, suggesting subtype 2 retinoblastoma was likely immune “cold” tumor (Fig. 5). We also noticed that the percentage of B cell lineage are invertedly correlated with the percentage of cytotoxic lymphocytes, monocytic lineage and myeloid dendritic cells in subtype 1a and 1b, suggesting different immunity (innate immunity in subtype 1a versus adaptive immunity in subtype 1b) approaches should be taken into consideration when we design immunotherapy for retinoblastoma.

For further investigating the inflammation occurred in the retinoblastoma, we then queried the cytokine profile across all subtypes of retinoblastoma. Interestingly, all subtypes of retinoblastoma shared the similar cytokine profiles (Supplementary Fig. 2).

### Subtype 1b retinoblastoma had a high risk of tumor invasion and prognosis

**Figure 6:**
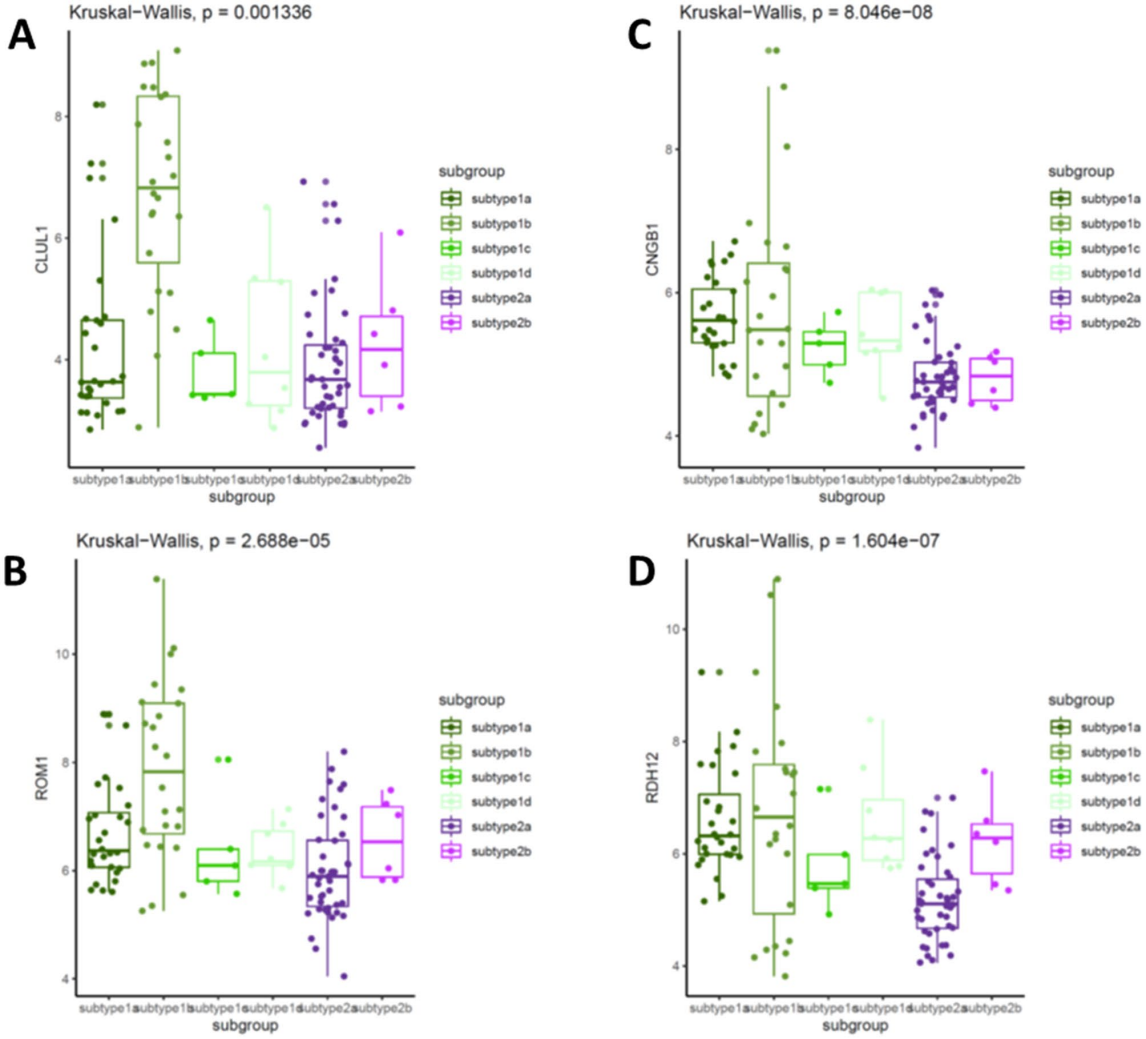
Invasive feature between subtypes. Boxplots showing the expression level of the invasive gene panel of CLUL1 (**A**), CNGB1 (**B**), ROM1 (**C**) and RDH12 (**D**) across subtypes.

Lastly, we examined whether the biological subtyping is indicated of the patients’ outcomes. Then, we compared the expression of retinoblastoma invasive biomarkers between subtypes. Based on our previous study, we have successfully identified a few retinoblastomas invasive signatures, providing the risk of tumor invasion. Interestingly, the retinoblastoma invasion markers (CLUL1, CNGB1, ROM1 and RDH12) were predominantly higher in subtype 1b, suggesting subtype 1b retinoblastoma prone to be more invasive as compared with other subtypes (Fig. 6). Kaplan–Meier Curve of patients’ overall survival also demonstrated that subtype 1b patients had worse outcomes while subtype 2a and 2b patients had relatively better outcomes (Log-rank test, p < 0.0001) (Supplementary Fig. 2).

## Discussion

Retinoblastoma is a frequent intraocular malignancy in children. Utilizing the gene expression profiling provides us an insight on the molecular heterogeneity in cancer biology^24,25^. In this study, we identified two major molecular subtypes and six minor subgroups with distinct profiles of tissue-of-origin marker. Subtype 1 retinoblastoma likely arise from cone as it usually has a higher expression of cone markers and Photoreceptor genes (such as ARR3 and GUCA1B). In contrast, subtype 2 retinoblastoma has a high expression of ganglion markers though some of cone markers have moderate levels, suggesting retinoblastoma from this subtype likely has less cone differentiation and contains neuronal or ganglion. Notably, subtype 2a has the highest expression of ganglion markers of all subgroups. Accumulating evidence show that expression of neuronal or ganglion genes are found in some types of cancer other than brain^26^. These cancers expressing neuronal genes are usually associated with tumor cell proliferation and migration^26^. Therefore, subtype 2 retinoblastoma is probably linked with the malignancy of retinoblastoma.

Previous studies have shown that stemness feature in retinoblastoma is associated with the metastasis and retinoblastoma with stemness feature is usually seen in cone-neuronal subtype^13^. In this study, we find that stemness index is significantly higher in subtype 2 retinoblastoma with higher expression of ganglion/neuronal markers. In addition, we also noticed that a higher stemness index in subtype 2 might be related to the decrease of RBL2. A reversely correlation of stemness index with the expression of RBL2 and TP53 and positive correlation in MDM4 in subtype 1 retinoblastoma, highlighting the MDM4 and TP53 likely have distinct function between subtypes^27,28^. Consistent with the previous findings, retinal cell type markers (such as SOX11, STMN2 and DCX) are also significantly highly expressed in subtype 2 retinoblastoma^13^. These results confirm our hypothesis that subtype 2 retinoblastoma is a cone-neuronal subtype.

Transcriptomic difference between subtypes reflects the heterogeneity of genetics in RB. Although RB1 germline mutation can be identified in both subtypes and all subgroups, the frequency of RB1 mutation is higher in the subtype 1 than in the subtype 2. The RB1 gene mutation in retinoblastoma disrupts the normal functioning of the retinal cells, including both photoreceptor cells (rods and cones) and neuronal cells^29^. Loss of RB leads to a reduction of E2F-dependent apoptosis and MDM2 expression level and p53-mediated apoptosis^30^. Our results also demonstrate a lower expression level of MDM2 in subtype 1 as compared with that in subtype 2, suggesting that RB1-mutated RB has exclusively cone-neuronal development deficiency and the potential effectiveness of cone-specific signalling circuitry relevant agents.

In addition to the difference of cone and ganglion markers expression between subtypes, we also find that the enriched pathways between subtypes are distinct. Through mapping the Gene Ontology of the DEG, we find the major difference of pathways between retinoblastoma subtypes are the regulation of immune process. A large difference of immune infiltration profile is identified between subtypes. In subtype 1a and 1b, both innate and adaptive immune response are likely activated. The major difference between subtype 1a and 1b is the subtype 1a is largely relied on the B cell immune response while innate immunity is most of the immune response in subtype 1a. In contrast, subtype 2 seems to be the immune desert as compared with subtype 1, suggesting the immune cell therapy might not be effective in these subpopulations^31^. Mao et al. also identified two mutually exclusive immune cell infiltration patterns in two major molecular subtypes^32^, highlighting that different immune cell-based strategy would be considered in the management of retinoblastoma.

Invasion is the key feature of tumor progression in retinoblastoma and always leads to the dismal outcomes. We have successfully identified a few gene signature (CLUL1, CNGB1, ROM1 and RDH12) to predict the risk of invasion in retinoblastoma and therefore, in this study, we investigated the profiles of invasive risk panel between subtypes. Subtype 2 (2a and 2b) has the relatively lower expression of the risk signature, suggesting that subtype 2 retinoblastoma likely has the low risk of invasion. Interestingly, subtype 1b with the lowest stemness index has the highest score for invasive risk. That might be because most retinoblastoma are likely epithelial-like cell rather than mesenchymal-like cells. The link of cancer stem cells and cancer invasion is usually observed in the mesenchymal cancer stem cells^33^.

Although our study highlights the difference of RB regarding the intrinsic key signalling pathways, there are some limitations in our study. First, the sample size of our study is still relatively small. As we propose six subgroups and only a few samples are assigned into one certain subgroup, more samples are required to validate our results. Second, we utilized a microarray-based platform to profile the biology difference within RB comprehensively, but it might not be a cost-effective approach in the clinical practice due to it requires personnel who specialized in bioinformatics to analyse the results. Therefore, a PCR-based gene panel indicating of the risk of invasion would be a more appropriate way in the future. Third, although we have revealed that the association of biological subtyping with patients’ outcomes, the association with progression is not fully elucidated yet. A prospective study will help us answer this in the future prior to translating into the clinical practice.

In conclusion, we identify two major and six minor molecular subtypes in retinoblastoma with distinct biological features. The major difference between the two subtypes is the level of cone and ganglion expression. Subtype with higher ganglion expression has higher stemness feature but has lower immune cell infiltration. Subtype 1b retinoblastoma has the highest score of tumor invasion. Different therapeutic strategy for retinoblastoma would be applied in the future.

### Supplementary Information

Supplementary Legends.

Supplementary Figure 1.

Supplementary Figure 2.

Supplementary Figure 3.

Supplementary Table 1.

Supplementary Table 2.

Supplementary Table 3.

## Data Availability

The raw microarray data has been deposited in GEO (GSE229598).
