# Supplementary material for: Transcriptome analysis reveals molecularly distinct subtypes in retinoblastoma
Source: Sci Rep. 2023 Sep 30;13:16475. doi: 10.1038/s41598-023-42253-4 (PMC10542806; doi:10.1038/s41598-023-42253-4)
Supplement: Supplementary file 1 — Supplementary Legends. [file 41598_2023_42253_MOESM1_ESM.docx]

# Supplementary Materials

Supplementary figure 1. Clustering of retinoblastoma. A, PCA plot showing the major difference between samples. B, Consensus matrix plot showing there are two major and six minor subtypes in retinoblastoma. C, Tracking plot showing the cluster number (from k= 2 to 10).

Supplementary figure 2. Cytokine expression between subtypes.

Supplementary figure 3. Kaplan-Meier Curve showing the overall survival (OS) of all included patients with different subtypes.

Supplementary Table 1. Clinical information of 114 retinoblastoma patients.

Supplementary Table 2. Differentially expressed genes between subtype 1 and 2.

Supplementary Table 3. Characteristics of patients clustered by molecular subtypes
