# Supplementary figures and images for: Transcriptome analysis reveals molecularly distinct subtypes in retinoblastoma
Source: Sci Rep. 2023 Sep 30;13:16475. doi: 10.1038/s41598-023-42253-4 (PMC10542806; doi:10.1038/s41598-023-42253-4)

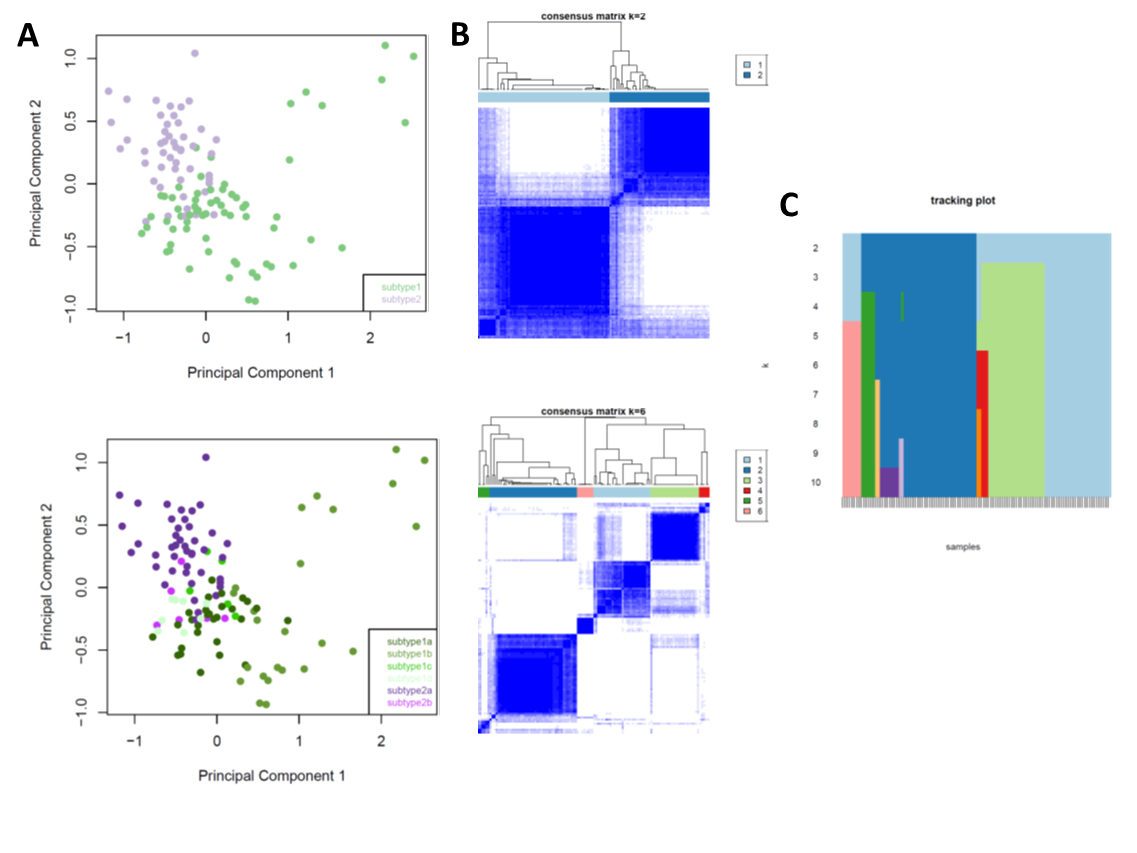

Supplement: Supplementary file 2 — Supplementary Figure 1. [file 41598_2023_42253_MOESM2_ESM.tif]

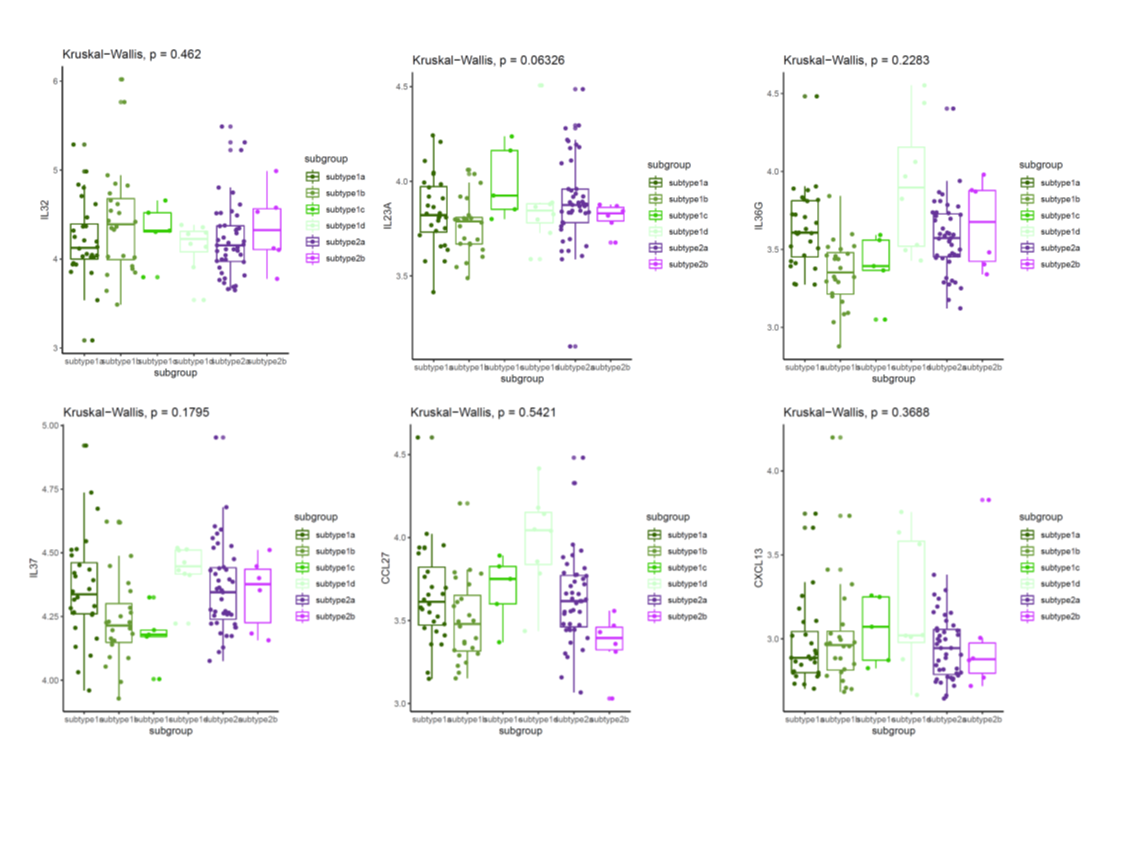

Supplement: Supplementary file 3 — Supplementary Figure 2. [file 41598_2023_42253_MOESM3_ESM.tif]

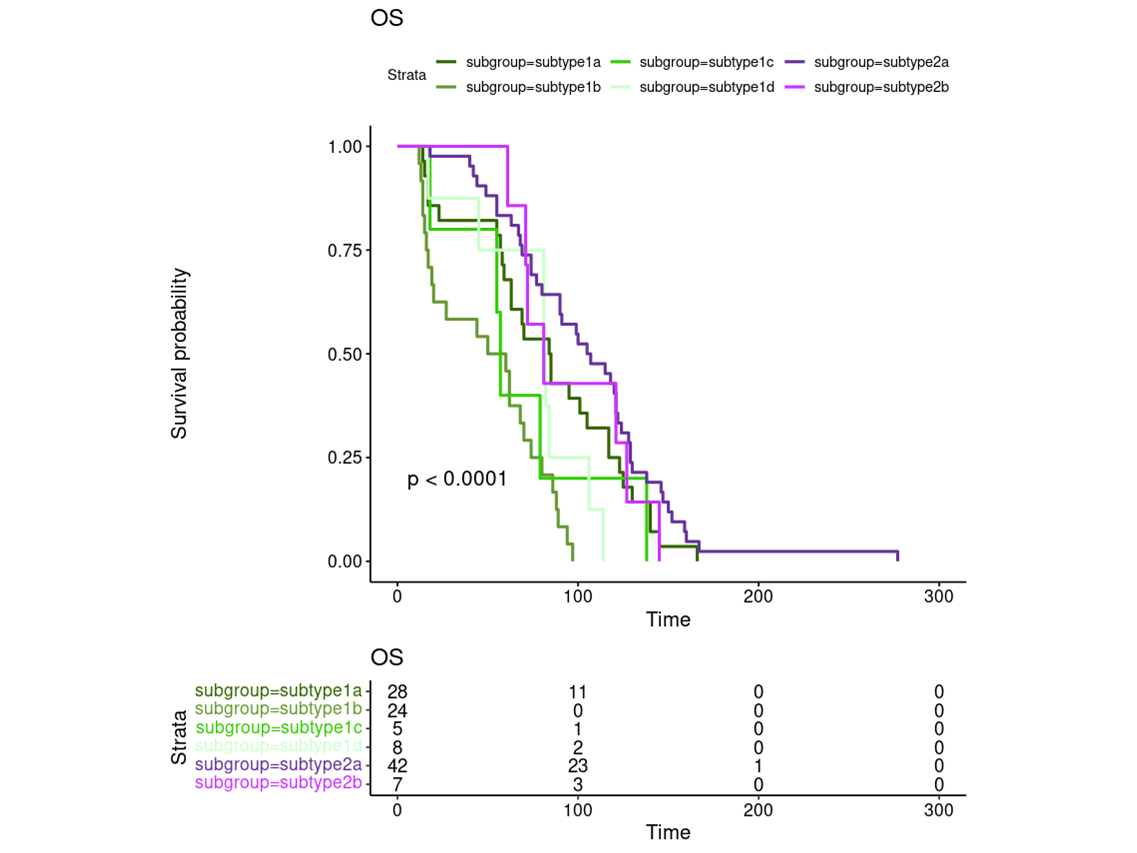

Supplement: Supplementary file 4 — Supplementary Figure 3. [file 41598_2023_42253_MOESM4_ESM.tif]
